# Effects of pelvic floor muscle training on sexual function of postmenopausal women. A systematic review and meta-analysis

**DOI:** 10.1093/sexmed/qfaf067

**Published:** 2025-09-19

**Authors:** Raquel García-Laria, Alejandra Alonso-Calvete, Lorenzo Justo-Cousiño, Iria Da Cuña-Carrera, Mercedes Soto-González

**Affiliations:** Faculty of Physiotherapy, University of Vigo, Vigo, PC 36001, Spain; Faculty of Physiotherapy, University of Vigo, Vigo, PC 36001, Spain; Faculty of Physiotherapy, University of Vigo, Vigo, PC 36001, Spain; FS1 Clinic Physiotherapy Group, Galicia Sur Health Research Institute, Vigo, PC 36312, Spain; Faculty of Physiotherapy, University of Vigo, Vigo, PC 36001, Spain; FS1 Clinic Physiotherapy Group, Galicia Sur Health Research Institute, Vigo, PC 36312, Spain; Faculty of Physiotherapy, University of Vigo, Vigo, PC 36001, Spain; FS1 Clinic Physiotherapy Group, Galicia Sur Health Research Institute, Vigo, PC 36312, Spain

**Keywords:** postmenopause, pelvic floor muscle training, physiotherapy, sexual dysfunction, Kegel exercises

## Abstract

**Background:**

During postmenopause, women frequently experience genitourinary symptoms that may result in sexual dysfunctions. Common treatments include hormone replacement therapy or vaginal lubricants. Pelvic floor muscle training (PFMT) has been observed to have beneficial effects on sexual function in other groups of women.

**Aim:**

To evaluate the effect of PFMT on sexual function in postmenopausal women.

**Methods:**

A systematic search was conducted in June 2025, in PubMed, Scopus, Web of Science, Medline, CINAHL databases, and the Google Scholar search engine. Inclusion criteria were randomized clinical trial articles published in English, in which at least one intervention addressed the study objective. A meta-analysis was conducted with a random-effects model.

**Results:**

A total of five studies were selected after applying eligibility criteria. All included articles implemented PFMT interventions, showing improvements in sexual function as assessed by the Female Sexual Function Index. A significant positive effect was shown in the total score of Female Sexual Function Index in experimental group in comparison with control group (*P* < .001; standard mean difference [SMD] = 1.33; *I*^2^ = 92%). A significant positive effect was also demonstrated in orgasm domain (*P* < .001; SMD = 1.91; *I*^2^ = 97%), arousal domain (*P* < .001; SMD = 1.87; *I*^2^ = 96%), and satisfaction domain (*P* < .001; SMD = 2.16; *I*^2^ = 98%). A significant negative effect was found in desire domain (*P* < .001; SMD = 0.34; *I*^2^ = 86%) and lubrication domain (*P* < .001; SMD = 0.26; *I*^2^ = 87%) and finally no significant effects were found in pain domain.

**Strengths and Limitations:**

Although this is the first meta-analysis to address this topic in postmenopausal women, the results are heterogeneous and the scientific evidence remains limited.

**Conclusion:**

PFMT appears to have positive effects on sexual function in postmenopausal women, particularly in aspects such as orgasm, arousal, and satisfaction.

## Introduction

Menopause is defined as the permanent cessation of menstrual bleeding, diagnosed after 12 months of amenorrhea, indicating the end of ovarian follicular activity. This stage symbolizes the end of a woman’s reproductive cycle, generally occurring between the ages of 45 and 55 years.^1^

Postmenopause refers to the phase immediately following menopause.^2^ During this period, women often experience genitourinary symptoms related to estrogen deficiency, leading to decreased vaginal epithelial thickness, trophism, vascularization, and lubrication.^3^ All these changes can lead to urogenital atrophy, vaginal dryness and itching, dyspareunia, pollakiuria, and urinary incontinence, among others, resulting in sexual dysfunctions.^4^

As for sexual dysfunctions in women, these are defined as an alteration in desire, arousal, orgasm, or the presence of pain during intercourse.^5^ Optimal sexual function has been characterized simply by the absence of dysfunction, which is far from reality since female sexual function, understood from a biopsychosocial approach, is influenced by biological, psychological, cultural, and social factors.^6^

It is estimated that, by 2025, the number of menopausal women will reach 1.1 billion worldwide.^7^ Although the incidence of sexual dysfunction after menopause is very uneven, varying from 19%^8^ to over 85%,^9^ we can observe that it is a highly prevalent problem in middle-aged women.

With regard to treatment, hormone replacement therapy (HRT) is the most effective option for the relief of genitourinary symptoms and, consequently, sexual dysfunction.^10^ Vaginal lubricants are also recommended as a non-hormonal agent.^11^

Another strategy that could be effective in the management of sexual dysfunction after menopause could be pelvic floor muscle training (PFMT), defined as an exercise program whose purpose is to improve the function and strength of the muscles that make up the pelvic floor through repeated voluntary contractions of varying duration and intensity.^12^ There are not many studies on the physiological mechanisms involved, but one of the most significant is by Shafik, which emphasizes the importance of the levator ani muscle in sexual function. During sexual intercourse, vaginal distension by the erect penis triggers the vagino-levator and vagino-puborectal reflexes, resulting in contraction of the levator ani muscle. This muscle also contracts in response to stimulation of the clitoris or the uterine cervix, an action mediated by the clitoromotor and cervicomotor reflexes. Contraction of the levator ani leads to dilation of the upper vagina, elevation and straightening of the uterus, and elongation and narrowing of the vaginal canal. These actions enhance the sexual response.^13^ Additionally, it has been demonstrated that the pubococcygeus muscle plays a key role in female orgasm, exhibiting rhythmic contractions induced by sexual arousal.^14^ Its weakness has been associated with anorgasmia, according to Graber and Kline-Graber.^15^

It is important to note that PFMT has reported beneficial effects on sexual function in other groups of women.^16-18^ In this regard, Bø et al. observed a statistically significant reduction in the number of women reporting problems with their sexual life after 6 months of PFMT, which involved performing 8 to 12 submaximal contractions in three daily sets.^19^ Jorge et al. conducted a systematic review and meta-analysis on PFMT in women with sexual dysfunction, concluding that PFMT significantly improved the total score of the Female Sexual Function Index (FSFI) as well as several subscales, including arousal, orgasm, satisfaction, and pain.^20^

Thus, it is hypothesized that this technique could also have a positive result during postmenopause. Therefore, the purpose of this study was to evaluate the effect of PFMT on sexual function in postmenopausal women through a review of the scientific literature. Specifically, we ask: Does PFMT improve sexual function in postmenopausal women?

## Methods

### Protocol and registration

We conducted a meta-analysis and systematic review to determine the effects of PFMT in the sexual function of women in the postmenopausal period. This study followed the PRISMA guideline (Preferred Reporting Items for Systematic Reviews and Meta-analyses)^21^ and was registered in the PROSPERO database (International Prospective Register of Systematic Reviews; CRD42024565537). The PRISMA checklist is available in Supplementary Material. This study used data from published articles and therefore did not require ethics committee review and approval.

### Eligibility criteria

Three researchers (R.G.L., M.S.G., I.D.C.) with practice experience in women’s health physiotherapy developed eligibility criteria. Inclusion criteria were randomized clinical trials, published in English and with at least one intervention related to the aim of this study, assessing changes in sexual function after PFMT in postmenopausal women.

### Search strategy

The search strategy was formulated based on the PICO research question (Patient, Intervention, Comparison, Outcome). The patient corresponds to postmenopausal women, the intervention is PFMT, and the outcomes are focus on sexual function. No comparisons were considered. Thus, the research question formulated to address is: *What effect does PFMT have on the sexual function of postmenopausal women?*

The systematic search was conducted in June 2025 using the databases PubMed, Scopus, Web of Science, Medline, and CINAHL. Additionally, a complementary search was performed using the Google Scholar search engine. The results were limited by language as specified in the inclusion criteria across all databases, while randomized clinical trials were manually filtered. Table 1 details the search strategies.

**Table 1:** Search strategy.

Databases	Search equations
PubMed	(“Menopause” [Mesh] OR “Postmenopause” [Mesh] OR “Perimenopause” [Mesh]) AND (“Sexual Behavior” [Mesh] OR “Sexuality” [Mesh] OR “Sexual Health” [Mesh] OR “sexual function” OR “sexual activity” OR “sexual*”) AND (“kegel” OR “pelvic floor muscle*” OR “exercise*”)
Scopus	TITLE-ABS-KEY (“menopaus*” OR “postmenopaus*” OR “perimenopaus*”) AND TITLE-ABS-KEY (“sexual behavior” OR “sexuality” OR “sexual health” OR “sexual function” OR “sexual activity” OR “sexual*”) AND TITLE-ABS-KEY (“kegel” OR “pelvic floor muscle*” OR “exercise*”)
Web of Science	((TS = (“menopaus*” OR “postmenopaus*” OR “perimenopaus*”))) AND TS = (“sexual behavior” OR “sexuality” OR “sexual health” OR “sexual function” OR “sexual*” OR “Sexual activity”) AND TS = (“kegel” OR “pelvic floor physical therapy” OR “pelvic floor muscle*” OR “exercise*”)
Medline	((MH “Menopause”) OR (MH “Postmenopause”) OR (“perimenopause”)) AND ((MH “Sexual Behavior”) OR (MH “Sexuality”) OR (MH “Sexual Health”) OR (“sexual function”) OR (“sexual activity”) OR (“sexual*”)) AND (“Kegel” OR “pelvic floor muscle*” OR “exercise*”)
Cinhal	((MH “Menopause”) OR (MH “Perimenopause”) OR (MH “Postmenopause”)) AND ((MH “Sexual Behavior”) OR (MH “Sexuality”) OR (MH “Sexual Health”) OR (“sexual function”) OR (“sexual activity”) OR (“sexual*”)) AND ((MH “Kegel Exercises”) OR (“pelvic floor muscle*”) OR (“exercise*”))
Google Scholar	((“postmenopause”) OR (“menopause”) OR (“perimenopause”)) AND ((“sexual function”) OR (“Sexual health”) OR (“sexual activity”) OR (“sexual”)) AND ((“pelvic floor muscle training”) OR (“Kegel”) OR (“exercise”))

ABS: Abstract; KEY: Keyword; Mesh: Medical Subjects Heading; MH: Medical Heading.

**Figure 1:**
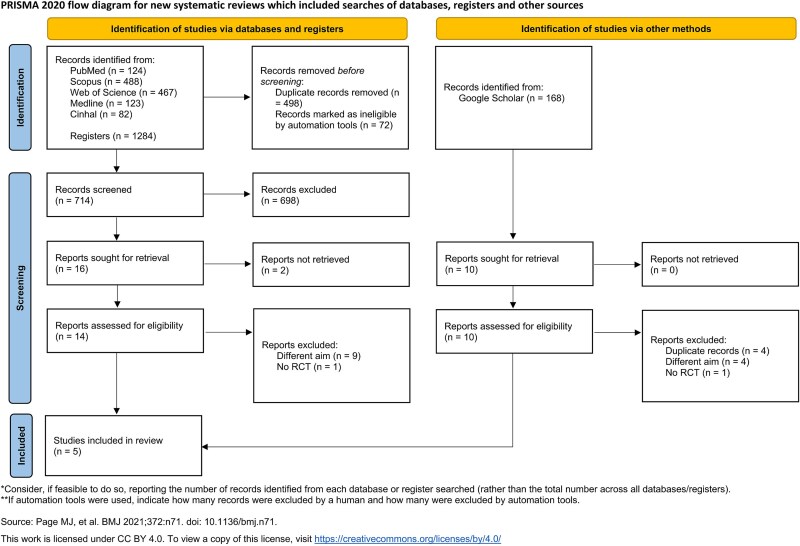
PRISMA flow chart.

### Selection process

The duplicates titles were removed with Zotero program (Center for History and New Media, George Mason University). An Excel file was used to screen titles and abstracts (R.G.L. and M.S.G.). Discrepancies were resolved through consensus. Full texts of potentially eligible studies were double screened independently (R.G.L. and I.D.C.) with discrepancies referred to a third researcher (A.A.C.).

### Data extraction

Data extraction included author names, publication year, country, number of participants, patients’ mean age, outcomes, interventions, and results. Data were compiled in an online spreadsheet accessible to all authors.

### Quality of studies

The methodological quality of the studies selected was assessed with the PEDro scale.^22^ This scale consists of 11 items, scoring 1 if the criteria is presented and 0 if it is not. The final score is from 0 to 10, since the first item does not count for the final score. Results <4 suggest a poor methodological quality; 4-5 a medium methodological quality; 6-8 a good methodological quality, and 9-10 an excellent methodological quality.^23^

Risk of bias was examined with “The Cochrane Collaboration” tool. This scale assess 6 items with “low risk,” “high risk,” or “unclear.”^24^

### Statistical analysis

The statistical analysis was conducted using Comprehensive Meta-Analysis software version 2.2.064 for Windows (Biostat Inc., Englewood, NJ, United States). Random effects models were conducted to determine the effects of PFMT on sexual function, including the total score of FSFI and each of the items of this test. In order to estimate the magnitude of PFMT intervention, standard mean differences (SMDs) values with 95% confidence intervals were used. The SMD were read as trivial (SMD < 0.2), small (0.2 ≥ SMD < 0.5), moderate (0.5 ≥ SMD < 0.8), or large (SMD ≥ 0.8).^25^ The significance level was set at *P* < .05 and heterogeneity was evaluated using the *I*^2^ statistic, to show the percentage of variation in estimated effects across studies, due to heterogeneity rather than chance. This *I*^2^ was interpreted as low (*I*^2^ < 25%), moderate (25% ≥ *I*^2^ < 75%), and high (*I*^2^ ≥ 75%).^26^

## Results

### Study selection

After the systematic search, 1452 studies were found. Eligibility criteria were applied and finally five studies were selected for this systematic review and meta-analysis, all of them Randomized Control Trial (RCT).^27-31^  Figure 1 details the search procedure and study selection.

Table 2 shows the main characteristics of the subjects.^27-31^ In total, 527 women participated in the study, with a sample size range between 40^28^ and 156^29^ subjects (mean age: 54 years old). All investigations, except from Khosravi et al.,^31^ reported losses to follow-up, ranging from 6.7% in the study by Nazarpour et al.^30^ to 15% in the study by Gonzaga et al.,^28^ with 58.06% of the attrition attributed to participant withdrawal.^27-30^

**Table 2:** Characteristics of the participants and eligibility criteria.

Study	Sample	Age (years)	Losses	Inclusion criteria	Exclusion criteria
Gonzaga et al.^28^	*N* = 40 IG1 = 20 IG2 = 20	IG1 = 56.10 (±5.26) IG2 = 61.40 (±5.08)	IG1 = 3 IG2 = 3	Women between 50 and 70 years old. Naturally postmenopausal (at least 1 year without menstruation). Be independent in activities of daily living. Effort UI.	Hysterectomy or oophorectomy. Cancer treatment with hormonal therapy. Cognitive impairment or neurological diseases. Regular physical exercise in the last 6 months. Presence of any severe alteration in the neuromusculoskeletal system. Inability to contract the pelvic floor muscles (Oxford scale <1). Pelvic organ prolapse >grade II according to Baden-Walker. Presence of urinary tract infection symptoms at the time of assessment. Previous participation in pelvic floor re-education programs.
Franco et al.^27^	*N* = 77 IG = 40 GC = 37	CG = 53.42 (±4.13) IG = 52.68 (±3.93)	IG = 3 CG = 4	A maximum of 5 years postmenopausal period. Not using HRT or using systemic HRT for more than 3 months. Having penetrative sexual intercourse in the last 4 weeks. Being able to perform a pelvic floor muscle contraction (≥1 according to the modified Oxford Scale). Being in a stable relationship for at least 4 months. Prolapse ≤grade I.	Women who experienced intolerance (pain or other discomfort) during the assessment. Participants who refused to answer the questionnaires.
Nazarpour et al.^29^	*N* = 156 IG1 = 52 IG2 = 52 GC = 52	IG1 = 51.5 (±3.4) IG2 = 53.1 (±2.7) CG = 52.9 (±4.0)	IG1 = 5 IG2 = 4 CG = 2	Natural menopause (not surgical or premature). Menopause occurring in the last 3 years. Being married and sexually active. No history of severe cardiovascular or mental disorders. Absence of sexual dysfunction in the spouse. Spouse with no history of drug addiction. Not undergoing HRT (hormone replacement therapy) or using medicinal herbs. Willingness to participate in the study.	Having debilitating diseases (eg, cancer) that prevent intervention. Undergoing any type of surgery. Experiencing marital discord. Being in the process of divorce. Death or illness of the spouse during the study period.
Nazarpour et al.^30^	*N* = 104 IG = 52 GC = 52	IG = 53.13 (±2.67) CG = 52.84 (±3.99)	IG = 5 CG = 2	Natural menopause (not surgical or premature). Menopause occurring in the last 3 years. Being married and sexually active. No history of severe cardiovascular or mental disorders. Absence of severe psychological distress in the last 3 months. Absence of sexual dysfunction in the spouse. Spouse with no history of drug addiction. Not undergoing chemical HRT or using medicinal herbs. Willingness to participate in the study.	Not willing to continue cooperating in the study. Having debilitating diseases (eg, cancer) that prevent the intervention. Undergoing any type of surgery. Experiencing marital discord. Being in the process of divorce. Death or illness of the spouse during the study period.
Khosravi et al.^31^	*N* = 150 IG1 = 50 IG2 = 50 GC = 50	IG1 = 52.2 (±3.27) IG2 = 54.22 (±2.56) CG = 55.28 (±3.3)	IG1 = 0 IG2 = 0 CG = 0	Physiological menopause. Being within the first 10 years of menopause. Absence of self-reported diseases affecting sexual function. Absence of self-reported urological or psychological diseases. Not suffering from depression, anxiety, or stress according to the Depression, Anxiety, and Stress Scales. Score ≥ 2 on pelvic floor muscle contraction according to the Oxford Scale. No pelvic organ prolapse. Being sexually active. Absence of sexual impotence in the spouse. Not undergoing chemical HRT or using medicinal herbs. Not taking any medication that affects sexual function. Absence of a history of previous pelvic surgery, hysterectomy, or mastectomy. A maximum of five vaginal deliveries. Absence of self-reported genital infection. BMI < 30.	Not willing to continue cooperating in the study. Allergy to the lubricating gel in the intervention group with gel.

Abbreviations: BMI: body mass index; CG: control group; IG: intervention group; IG1: intervention group 1; IG2: intervention group 2.

### Study characteristics

All included studies recruited postmenopausal women.^27-31^ The definition of the postmenopausal period varied, from at least 1 year without menstruation in Gonzaga et al.^28^ to 10 years in Khosravi et al.^31^ Additionally, most studies required participants to be sexually active and not undergoing HRT.^20^^,^^28-30^ Gonzaga et al.^27^ specified stress incontinence as an inclusion criterion, while some studies included scoring criteria based on the Oxford Scale^28^^,^^31^ or the Modified Oxford Scale.^27^ Common exclusion criteria comprised conditions or disorders preventing intervention.^28-31^ Furthermore, Gonzaga et al.^28^ and Khosravi et al.^31^ specified that participants should not have undergone gynecological,^27^ pelvic, or mastectomy surgeries.^30^

Table 3 details the interventions performed on the studies, as well as frequency of the treatment, duration, variables assessed, and results.

**Table 3:** Intervention, duration and frequency, variables, and outcomes of the articles.

Study	Intervention	Duration and frequency	Variables and instruments	Results
Gonzaga et al.^27^	IG1: PFMT. 4 sets of 10 maximal contractions (6 s) with equal rest time. At the end of each set, 5 rapid contractions. IG2: 10 Pilates exercises performed in a single set of 10 repetitions.	12 weeks. 3 sessions per week. 30 min/session	Sexual Function (FSFI)	No significant differences between groups. Intragroup: Intragroup: IG1: ↑ desire, ↑ lubrication, ↑ pain, ↑ orgasm. IG2: ↑ desire, ↑ lubrication.
Franco et al.^20^	IG: PFMT. 4 sets of 10 maximal contractions (6 s). At the end of each set, 5 rapid contractions.CG: No intervention	12 weeks. 1 session a day	Sexual Function (FSFI) Pelvic floor function (MOS)	More women with no dysfunction in IG. No differences between the groups regarding pelvic floor muscle function.
Nazarpour et al.^28^	IG1: Sexual education program (information about sexual function, sex during menopause, and lubricants) IG2: PFMT. Kegel exercises. CG: General education about menopause.	12 weeks. IG2: 1 session a week.	Sexual Function (FSFI)	Comparing with GC: IG1: ↑ arousal. IG2: ↑ arousal, ↑ orgasm, ↑ satisfaction.
Nazarpour et al.^29^	IG: PFMT. 10 contractions of 10 s with 10 s of rest. Only 5 s of contraction if women had difficulties with 10 s. CG: General education about menopause.	12 weeks IG: 3-4 sessions a day.	Sexual Function (FSFI)	IG: ↑ arousal, ↑ orgasm, ↑ satisfaction.
Khosravi et al.^30^	IG1: PFMT. Kegel exercises IG2: Lubricants during sex CG: No intervention	12 weeks IG1: 30 repetitions. 3 sessions a day.	Sexual Function (FSFI)	IG1 and IG2 showed significant improvement in all FSFI domains in comparison with CG. An improved sexual function is more likely in IG1 in comparison with IG2

Abbreviations: CG: control group; FSFI: Female Sexual Function Index; IG: Intervention group; IG1: Intervention group 1; IG2: Intervention group 2; MOS: Modified Oxford Scale; PFMT: Pelvic floor muscle training.

To compare treatments, each sample was divided into control and one^27^^,^^30^ or two^28^^,^^29^^,^^31^ intervention groups. All studies employed PFMT. Three studies provided specific details about the exercise characteristics, including sets, repetitions, and rest intervals,^27^^,^^28^^,^^30^ while the remaining studies did not provide such details.^29^^,^^31^

The interventions lasted 12 weeks in all studies.^27-31^ However, the exercise frequency varied, with daily sessions,^27^^,^^29^ three daily sessions,^30^^,^^31^ or three weekly sessions. Sessions were individualized and supervised in the study of Gonzaga et al.,^28^ or supervised twice weekly in Franco et al.^27^ The remaining studies used weekly telephone follow-ups.^29-31^

All studies assessed sexual function as the primary outcome,^27-31^ using the FSFI, a brief multidimensional scale comprising 19 items across six domains: sexual desire, arousal, lubrication, orgasm, satisfaction, and pain.^32^ The results were summarized according to these six domains, in order to improve understanding. Additionally, Franco et al.^27^ evaluated PFMT using the Modified Oxford Scale, which scores muscle strength from 0 to 5.^33^ In all studies, the whole sexual function has been measured, and no specific domain has been evaluated after a PFMT intervention.

Educational sessions for PFMT groups were conducted in all studies, provided individually^27^^,^^28^^,^^31^ or in groups.^29^^,^^30^ These sessions were conducted during the first week,^28^ the initial assessment,^27^ or before starting the 12-week intervention.^29-31^

### Meta-analysis and statistical results

**Figure 2:**
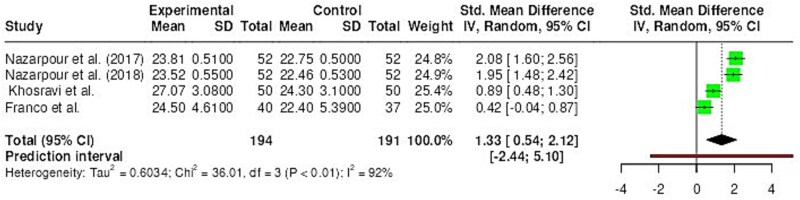
Total score of FSFI.

Results of the meta-analysis regarding the total score of FSFI are detailed in Figure 2. These results show a significant positive effect on the experimental group in comparison with the control group (*P* < .001; SMD = 1.33 (large); *I*^2^ = 92%, high).

**Figure 3:**
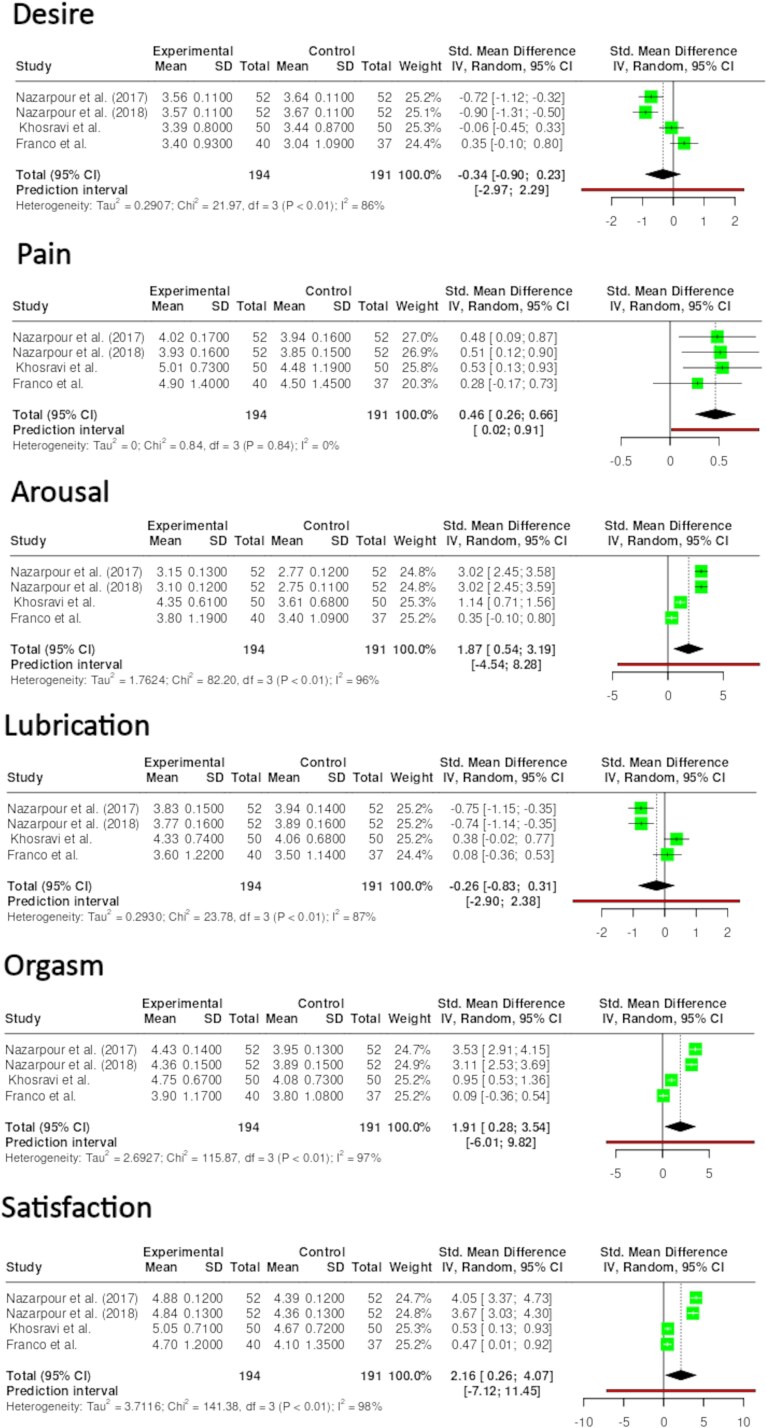
Score of the domains of FSFI.

Figure 3 presents domain-specific results. Significant positive effects were observed in the experimental group for orgasm (*P* < .001; SMD = 1.91; *I*^2^ = 97%), arousal (*P* < .001; SMD = 1.87; *I*^2^ = 96%), and satisfaction (*P* < .001; SMD = 2.16; *I*^2^ = 98%) domains of the FSFI. Significant negative but small effects were found for desire (*P* < .001; SMD = 0.34; *I*^2^ = 86%) and lubrication (*P* < .001; SMD = 0.26; *I*^2^ = 87%), while no significant effects were observed for pain (*P* > .05).

### Methodological quality and risk of bias

The methodological quality of the selected articles,^27-31^ assessed using the PEDro scale,^22^ is detailed in Table 4. Three articles achieved good methodological quality, scoring 6^28^^,^^31^ and 7,^27^ while the remaining two articles were of medium quality with scores of 5.^29^^,^^30^

**Table 4:** Results of the PEDro scale.

Study	1	2	3	4	5	6	7	8	9	10	11	Total
Gonzaga et al.^30^	1	1	1	1	0	0	0	0	1	1	1	6
Franco et al.^27^	1	1	1	1	0	0	1	1	0	1	1	7
Nazarpour et al.^30^	1	1	0	1	0	0	0	1	0	1	1	5
Nazarpour et al.^30^	1	1	0	1	0	0	0	1	0	1	1	5
Khosravi et al.^31^	1	1	0	1	0	0	0	1	1	1	1	6

0: No; 1: Yes; Item 1: Eligibility criteria were specified. Item 2: Subjects were randomly allocated to groups. Item 3: Allocation was concealed. Item 4: Groups were similar at baseline regarding the most important prognostic indicators. Item 5: All subjects were blinded. Item 6: All therapists who administered the therapy were blinded. Item 7: All assessors who measured at least one key outcome were blinded. Item 8: Measurements for at least one key outcome were obtained from more than 85% of the subjects initially allocated to groups. Item 9: Results were reported for all subjects who received treatment or were allocated to a control group, or when this was not possible, data for at least one key outcome were analyzed by intention-to-treat. Item 10: Statistical comparisons between groups were reported for at least one key outcome. Item 11: The study provides point measures and measures of variability for at least one key outcome.

**Figure 4:**
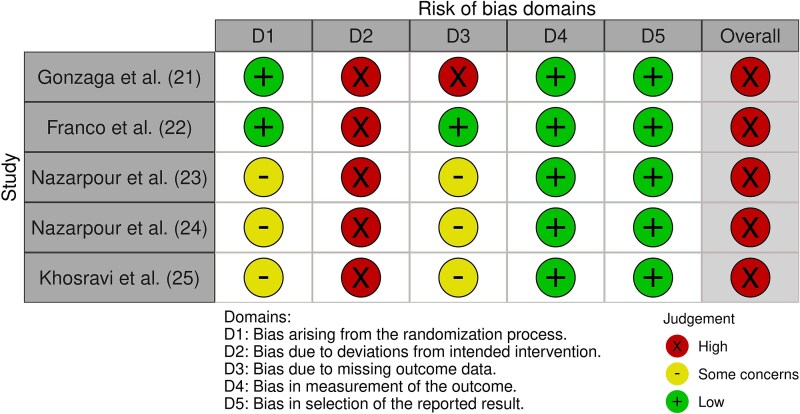
Results of the risk of bias.

About the risk of bias evaluation, it is shown in Figure 4. All studies demonstrated low risk in sequence generation^27-31^ but only Gonzaga et al.^27^ and Franco et al.^27^ reported low risk in allocation concealment, while this was unclear in the other studies.^27-31^

Performance bias was high across all studies.^27-31^ Only Franco et al.^27^ employed blinded assessors, resulting in high risk in Gonzaga et al.^28^ and unclear risk in the other studies.^29-31^ All research reported complete outcomes,^27-31^ with selective reporting observed in Gonzaga et al.,^28^ Franco et al.,^27^ and Khosravi et al.,^31^ while high risk of reporting bias was noted in Nazarpour et al.^29^^,^^30^ Finally, no other sources of bias were identified.

## Discussion

The aim of the present study was to determine the effects of PFMT on sexual function in postmenopausal women. In general terms, we found that this technique has shown positive effects, although the evidence found is limited. Other previous reviews, although not focused on postmenopausal women, show improvements in sexual function with PFMT^34^ and suggest that PFMT may prevent pelvic floor dysfunction and improve sexual function, especially in women with underlying pathologies.^35^

The results of the meta-analysis show a large positive effect with PFMT on female sexual function measured with the FSFI compared to the control group. However, the improvements are not homogeneous in terms of dimensions of the FSFI, finding differences between the investigations discussed below.

**Orgasm** is a brief and variable peak of intense pleasure involving involuntary rhythmic contractions of the pelvic floor muscles, often accompanied by uterine and anal contractions and muscle tension.^36^

The meta-analysis shows large positive effects on orgasm. In agreement with the findings, some authors show beneficial results in this domain in women with overactive bladder^37^ and during postpartum.^38^

This fact is consistent since, among the great diversity of factors influencing orgasm, we find adequate Pelvic Floor Muscle (PFM) strength,^39^ so that weakness has a negative impact on orgasm capacity. Therefore, strengthening the PFM would increase orgasmic capacity, which has been corroborated by several authors who have shown greater orgasm in women with greater PFM strength compared to those with weakness.^40-42^

However, other researchers have shown that cognitive-affective factors, such as thoughts and emotions, can significantly interfere with the orgasmic response of women,^43^ so that PFMT may not be sufficient to obtain benefits.

***Arousal***, on the other hand, is subdivided into subjective and genital arousal.^44^ The meta-analysis shows large positive effects in this domain. Although subjective arousal is influenced by a wide variety of factors on which PFMT has no impact, such as feeling desired by the sexual partner or a negative mood^45^; we can hypothesize that its improvement could be produced by greater control and awareness of the pelvic floor, accompanied by a decrease in pain, greater lubrication, and, in general, more satisfying and positive experiences for the woman, promoting a greater connection with her body during sexual activity.

The ***satisfaction*** dimension is understood as the affective response that arises from a subjective evaluation of the positive and negative factors associated with one’s own sexual activity.^46^ The FSFI scale includes three items that assess satisfaction, two of which refer to the partner and the third to sexual life in general.^47^

It has been shown that sexual satisfaction is closely related to orgasm.^48^ As we have previously observed, orgasmic capacity is improved by means of PFMT, so that reducing it to this factor could explain the increase in this domain. This is consistent with the findings of our meta-analysis, where positive results are found. Furthermore, in the three studies where an improvement in satisfaction was observed, an increase in orgasm was also recorded.^29-31^ Improvement in other aspects such as lubrication and decreased pain is equally necessary elements for satisfactory activity, although no positive results have been obtained in these domains separately. This, in turn, contributes to increased confidence and self-esteem, factors that according to Antičević et al.^49^ are linked to sexual satisfaction. In reality, this domain encompasses a broader concept, where factors such as mental health, communication or satisfaction with the couple’s relationship beyond the sexual domain also play a role.^50^

***Desire*** is defined as the impulse or motivation that leads an individual to participate and/or be receptive to sexual activity.^51^ In line with Zahariou et al.^52^ and Schütze et al.,^38^ four of the studies found significantly positive results for this domain.^28-31^ However, the meta-analysis showed a significant negative result for this domain compared to the control group and with a small effect. Desire is not an isolated fact, in which many other factors intervene, and this may explain the difference between the results.^51^

The ***lubrication*** domain reflects genital arousal^47^ and involves fluid production that protects against friction, along with genital vasocongestion and increased sensitivity during sexual stimulation.^51^^,^^53^^,^^54^

The effects of PFMT on this domain are controversial, so that according to the existing literature, while some studies show no improvement,^37^ others,^55^ including two of those analyzed in this review,^28^^,^^31^ do show improvements. As with desire, the meta-analysis found a negative relationship, albeit a small one. Lubrication is closely linked to hormone levels,^56^ a variable on which PFMT has no impact and which may explain the lack of improvement.

Finally, ***pain*** is described as an unpleasant experience involving sensory and emotional factors associated, or similar to that experienced, to potential or actual tissue damage.^57^ The FSFI scale refers to pain experienced both during and after sexual intercourse.^47^

According to Mercier et al.,^58^ PFMT has shown very positive results in terms of the vulvovaginal atrophy that occurs in this phase, which is largely responsible for the pain. This can be attributed to the fact that the benefits of PFMT include not only increased lubrication, but also improved tissue elasticity, trophism, and vascularization of the area,^59^ factors that contribute to the mitigation of vulvovaginal atrophy symptoms and thus, pain. At the same time, PFMT may not address the underlying cause of pain or may not be sufficient. The above relationships could explain why we found no effect on this variable in our meta-analysis.

In relation to the studies that make up this review, all the interventions have a duration of 12 weeks.^27-31^ Aukee et al.^60^ demonstrated that, after 12 weeks of PFMT, pelvic floor muscle activity values increased significantly. The above provides evidence of the suitability of this duration for obtaining results, supporting its use as the established time in most studies. Likewise, this does not imply that it is the minimum time required, since, in the article by Khosravi et al.,^31^ reevaluations were carried out every 4 weeks, showing beneficial results from the first measurement.

With regard to the exercise program and the frequency with which they should be performed, there are various options: one daily session^27^ or three weekly sessions^28^ of 4 series of 10 maximum contractions of 6,“with equal rest time, followed by 5 rapid contractions at the end of each series or three daily sessions of 30′ in which 10 contractions of 10” are performed, with equal rest time.^29^

Originally, Kegel described that the exercises should be performed 20′, three times a day, reaching a total of 300 contractions.^61^ PFMT programs in the current scientific literature are varied, so that there is no single, standardized protocol.^62^ This could be attributed to the importance of individualization and adaptation of the parameters according to the circumstances of each woman, which could possibly allow us to achieve better results.^63^ The remaining studies do not reflect the exercise program, showing only the frequency used in the intervention.^29^^,^^31^ It is crucial to specify the parameters in order to ensure reproducibility and transparency in the research.

Regarding supervision during exercise, the review by Kharaji et al.^64^ showed that accompaniment by a professional led to significantly greater improvements. In contrast, in the articles included in this review, the presence^27^^,^^28^ or absence^29-31^ of this variable had no impact on obtaining equally positive results.

This could suggest that the correct instruction of the exercises by a professional would allow the women to work autonomously, achieving beneficial results, despite the fact that, according to the aforementioned review, those obtained with direct supervision are superior.^64^

Four of the included studies mentioned not being subjected to HRT^27^^,^^29-31^; however, the research by Gonzaga et al.^28^ does not mention anything about HRT. According to the recent review by Meziou et al.,^65^ it has been observed that this therapy can slightly improve sexual function in peri- and postmenopausal women. Therefore, the effects of HRT can interfere with the results of the studies, so that establishing women not undergoing this therapy as an inclusion criterion would allow a more specific and isolated evaluation of the effects of PFMT, facilitating more precise conclusions.

Furthermore, as initially mentioned, four of the studies include women in the postmenopausal period with urinary incontinence (UI),^27-29^ and specifically, in the article by Gonzaga et al.,^28^ the entire sample presents this condition.

This is relevant because urine leakage can frequently occur during sexual activity.^66^ Therefore, it can be hypothesized that the results in these women could be more positive since, by strengthening the PFM, it is possible to increase continence, reducing the probability of occurrence of these episodes and mitigating the fear of urinating, which, in turn, would increase confidence. These benefits would result in the improvement of aspects that negatively influence sexual function.

Regarding the risk of bias, the studies present a high level of performance bias and therefore limitations in terms of blinding of the participants and therapists. This is very common in physical therapy, since it involves direct interventions. Only the article by Franco et al.^27^ shows a low risk of blinding of the evaluator. This is relevant because knowledge of the participants’ assignment to the different intervention groups can consciously or unconsciously influence the way the data are interpreted, affecting the objectivity of the evaluation and thus compromising the results.

A limitation for the studies is the lack of clarity regarding the concept of “being sexually active,” which is reflected as an inclusion criterion in three articles.^29-31^ According to Khosravi et al.,^31^ this concept is subject to the participants’ personal interpretation, while Nazarpour et al.^29^^,^^30^ establish it as an inclusion criterion without specifying the type of sexual activity to which it refers.

The FSFI does not refer exclusively to vaginal intercourse, but also includes self-stimulation and sexual games or fantasies. This lack of clarity could lead to misinterpretation by the participants, who may consider that it refers exclusively to penetrative sexual activity. Thus, all of the above could condition the responses and, consequently, the results of the studies.

In addition to the above, Franco et al.^27^ included only penetrative intercourse, which would be inadequate, considering that this is not the only aspect of sexual function according to the FSFI.

Another limitation lies in the inclusion by Gonzaga et al.^28^ of seven non-sexually active women in their study, altering the results, since it is not possible to evaluate whether or not there are any improvements in the different domains.

It should also be noted that the questionnaires are completed with an interviewer^27-31^ in all the articles, with the exception of Khosravi et al.,^31^ where there is no specification in this regard. Sexuality is a more intimate and sensitive area in which the physical presence of another person may be uncomfortable or embarrassing for the participants, making them feel self-conscious, which could lead them to provide answers that are not completely honest.^67^

Therefore, self-administration of the questionnaire, with a previous explanation of the same, could encourage sincerity about the answers, allowing the participants to feel more comfortable.

It should also be noted that the lack of description of the exercise programs used in the studies means that these interventions cannot be reproduced, also compromising their transparency.

Finally, with regard to the limitations of the studies, it should be pointed out that Franco et al.^23^ established the absence of HRT as an inclusion criterion, but 30% of the participants were subjected to HRT. As previously explained, HRT has effects on the sexual function of women, so these effects may interfere with the results of the studies.

As a limitation of this review, it is important to highlight the limited scientific evidence available in this population, together with the presence of small sample sizes and limited methodological quality.

Therefore, as future lines of study, we propose to carry out more research in this population to better understand the effects of PFMT on sexual function, as well as larger and more homogeneous samples, in order to obtain more representative and generalizable results, together with better methodological quality.

## Conclusion

After the meta-analysis of the articles included in this review, it could be concluded that PFMT appears to have positive effects on sexual function in postmenopausal women, especially on orgasm, arousal, and satisfaction.

However, it has been observed that sexual function is a biopsychosocial phenomenon, not only influenced by physical factors, and therefore the results should be interpreted taking these variables into consideration. Nevertheless, these results should be taken with caution due to the paucity of existing literature in this area. The results should be interpreted with caution due to the study’s limitations and the high heterogeneity of the reviewed studies. Therefore, additional, more specific studies are required in future research.

## Supplementary Material

PRISMA_CHECK_LIST_20_7_qfaf067
